# Understanding and Managing Pregnancy in Patients with Lupus

**DOI:** 10.1155/2015/943490

**Published:** 2015-07-12

**Authors:** Guilherme Ramires de Jesus, Claudia Mendoza-Pinto, Nilson Ramires de Jesus, Flávia Cunha dos Santos, Evandro Mendes Klumb, Mario García Carrasco, Roger Abramino Levy

**Affiliations:** ^1^Department of Obstetrics, Universidade do Estado do Rio de Janeiro, Rio de Janeiro, Brazil; ^2^Systemic Autoimmune Diseases Research Unit, Hospital General Regional No. 36-CIBIOR, Instituto Mexicano del Seguro Social, Puebla, Mexico; ^3^Department of Immunology and Rheumatology, Medicine School, Benemérita Universidad Autónoma de Puebla, Puebla, Mexico; ^4^Department of Rheumatology, Universidade do Estado do Rio de Janeiro, Rio de Janeiro, Brazil

## Abstract

Systemic lupus erythematosus (SLE) is a chronic, multisystemic autoimmune disease that occurs predominantly in women of fertile age. The association of SLE and pregnancy, mainly with active disease and especially with nephritis, has poorer pregnancy outcomes, with increased frequency of preeclampsia, fetal loss, prematurity, growth restriction, and newborns small for gestational age. Therefore, SLE pregnancies are considered high risk condition, should be monitored frequently during pregnancy and delivery should occur in a controlled setting. Pregnancy induces dramatic immune and neuroendocrine changes in the maternal body in order to protect the fetus from immunologic attack and these modifications can be affected by SLE. The risk of flares depends on the level of maternal disease activity in the 6–12 months before conception and is higher in women with repeated flares before conception, in those who discontinue useful medications and in women with active glomerulonephritis at conception. It is a challenge to differentiate lupus nephritis from preeclampsia and, in this context, the angiogenic and antiangiogenic cytokines are promising. Prenatal care of pregnant patients with SLE requires close collaboration between rheumatologist and obstetrician. Planning pregnancy is essential to increase the probability of successful pregnancies.

## 1. Introduction

Systemic lupus erythematosus (SLE) is a chronic, multisystemic autoimmune disease that occurs predominantly in women of fertile age. The risk of obstetric complications in pregnant SLE patients is significant, with an increased risk of spontaneous abortion, intrauterine fetal death, preeclampsia (PE), intrauterine growth restriction (IUGR), and preterm birth. In addition, pregnancy may be associated with disease flares requiring immunosuppressive therapy. Therefore, pregnancies in SLE patients are considered a high risk condition. Maternal health and fetal development should be monitored frequently during pregnancy. If possible, delivery should occur in a controlled setting. An obstetrician with experience in high-risk pregnancies should follow pregnant women with SLE, including a multidisciplinary approach with rheumatologic and neonatal team. Fortunately, due to medical advances the number of SLE patients who become pregnant has increased worldwide and most pregnancies are successful [1, 2].

Although these patients have fewer live births with more pregnancy complications, they may have subsequent uncomplicated pregnancies after a poor outcome. Recent studies have analyzed novel markers of poor pregnancy outcomes and new approaches to the management of SLE during pregnancy and SLE activity during pregnancy remains an ongoing problem, since major organ involvement can negatively affect outcomes [3]. Adverse fetal outcomes in obstetric SLE include fetal loss (spontaneous abortion and intrauterine fetal death), IUGR, premature birth, premature rupture of membranes, neonatal lupus, and perinatal mortality. Maternal complications in SLE patients include SLE activity, PE, and arterial hypertension, especially in patients with renal involvement [4].

A recent population-based study by Vinet et al. followed 1334 women with SLE through the Quebec administrative databases and found that SLE women have fewer live births than the general population. Over a 9-year period, 559 live births occurred in SLE patients compared with the 708 that would have been expected in the general population (standardized incidence ratio 0.79; 95% confidence interval (CI) 0.73–0.86) [5].

In the United States, there are an estimated 4500 pregnancies in SLE women each year and pregnancy complications are common: one-third of the pregnancies result in a caesarean section, the birth is preterm in 33% of all gestations, and over 20% of all women will develop by PE [2].

Studies suggest that fetal loss may be decreasing in recent years. In 1960 to 1965, the mean rate of fetal loss was 43%, compared with 17% in 2000 to 2003 [4]. However, in a multiethnic population with SLE in North America, the fetal loss rate may be related to comorbidities and disease activity before pregnancy [6]. Thus, the risk of fetal loss is higher in women with hypertension, active SLE [7], lupus nephritis (LN) [8, 9], hypocomplementemia, elevated levels of anti-DNA antibodies, antiphospholipid antibodies (aPL), or thrombocytopenia [10, 11]. Further research is required to confirm this correlation in lupus pregnancy; several factors may predict fetal death, such as SLE activity, active LN, and the presence of aPL [6].

An optimistic finding from a population-based study in New South Wales, Australia, which looked at 675 women with SLE and 1058 deliveries, suggested that women whose first pregnancies result in perinatal death could nevertheless expect a live birth in subsequent pregnancies [12]. However, it is not clear whether parity increases the risk of SLE, as high-quality studies of large datasets have produced conflicting results [13].

## 2. Interaction of Pregnancy and Systemic Lupus Erythematosus

Pregnancy induces dramatic immune and neuroendocrine abnormalities in the maternal body in order to protect the foetus from immunologic attack by the mother. Instead of general immunosuppression, which would weaken maternal defense against infection, there is a modulation of the composition and function of immune-competent cells and immune-modulatory molecules in the maternal system during pregnancy [14]. The fetus promotes tolerance to paternal antigens by migration of fetal cells and cell-free fetal DNA to the maternal circulation during normal pregnancy. Fetal cells remain in the mother for decades, creating a state of microchimerism [15, 16].

**Figure 1:**
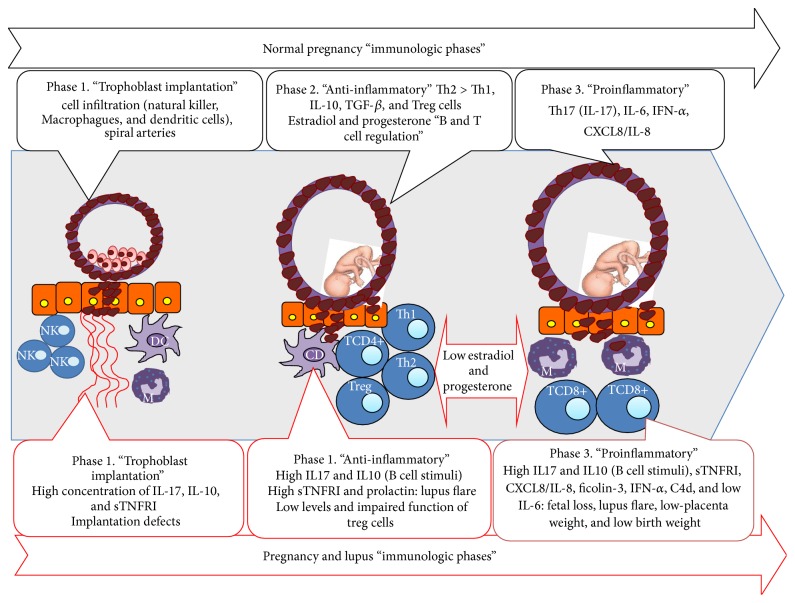
Pathogenesis in normal and SLE pregnancy.

After delivery, the maternal body adjusts to a nonpregnant state, which is not simply a return to the condition before conception, but occurs under the influence of immune activation at parturition [17]. The profound immunologic adaptations necessary for maternal tolerance of the foetus in pregnancy and the immunologic reset to a nonpregnant state thereafter, influence maternal autoimmune rheumatic diseases in several ways.  Figure 1 shows the main elements interacting in SLE and pregnancy.

The activity of immunocompetent cells is regulated by cytokines and chemokines with T helper cells as key effectors. Cytokines are important mediators acting in concert with other factors to support successful pregnancy. One of the most important immunological modifications during pregnancy is the Th1/Th2 cytokine shift. Th1 includes interferon (IFN)-*γ*, interleukin- (IL-) 1, IL-2, IL-12, and tumor necrosis factor- (TNF-) alpha, which stimulate cellular immunity, and Th2 includes IL-4, IL-5, IL-6, and IL-10, which induce humoral immunity and antibody production. Since SLE is mainly a Th2-mediated disease, during pregnancy a predominance of the Th2 response may be expected, making disease exacerbation more likely. However, lower levels of estrogens, progesterone, and Th2 cytokines were found in the third trimester of pregnancy in SLE patients compared with healthy pregnant women [18].

Besides Th1 and Th2 cells, there is a third subset of CD4+T helper cells, Th17 cells, which activate the immune system and interleukin-17 (IL-17) family [19–21]. IL-17 has a proinflammatory effect and drives the development of Th17 cells, whereas a milieu of tolerance promotes the development of Treg cells [19, 22]. Increased numbers of Th17 cells are also found in conditions linked to pregnancy, such as preeclampsia and recurrent pregnancy loss (Figure 1) [23, 24].

SLE is characterized by a loss of tolerance in both the T cell and B cell compartments, resulting in B cell hyperreactivity with pathogenic autoantibody formation. One important factor that has emerged is the response of SLE to sex steroid hormones [25]. Estrogens enhance antibody production, T helper type 2 immune responses, and B cell immunity. At high concentrations, such as those found in pregnancy, estrogens and gestagens stimulate the secretion of IL-4, IL10, TGF-*β*, and IFN-*γ* while simultaneously suppressing production of TNF-*α* [26].

In SLE patients, IL-6 serum levels remained low and did not increase in the third trimester of pregnancy, as they did in healthy controls. The absence of a rise in IL-6 is interesting in the context of cytokine function: IL-6 is necessary for T cell help for B cells [27]. IL-10 did rise progressively during pregnancy in healthy women [18]. In contrast, IL-10 levels were already significantly higher at conception in SLE patients and remained elevated throughout pregnancy and postpartum. IL-10 was originally thought to be a Th2 cytokine but is now known to be a pleiotropic cytokine with both immune stimulatory and immune suppressive functions that place it outside the Th1-Th2 paradigm [27]. Persistent high levels of IL-10 indicate a constitutional overproduction in SLE, resulting in continuous B cell stimulation.

No significant differences between SLE patients and controls were found in either sTNFR I or II levels or profiles before and during pregnancy. sTNFR I levels were significantly higher during pregnancy and postpartum in SLE patients with active disease compared with healthy controls [18]. Studies of regulatory T cells in SLE patients have shown reduced numbers in active SLE and impaired suppressive function of Treg cells [28]. A pilot study indicates that there is an imbalance between Treg and Th17 numbers in pregnant SLE patients [29].

During and after pregnancy, women with SLE have increased serum concentrations of CXCL8/IL-8, CXCL9/MIG, CXCL10/IP-10, and IL-10 chemokines compared with normal pregnancies, especially during active disease (Figure 1) [30].

Ficolins are soluble molecules of the innate immune system that recognize carbohydrate molecules on microbial pathogens and apoptotic and necrotic cells. Elevated ficolin-3 in specific manifestations may indicate a pathogenetic role of ficolin-3 in SLE. Ficolin-2 may be involved in the direct removal of trophoblast-derived material from the maternal circulation. Low levels of circulating ficolin-2 might impair the clearance of shed apoptotic and necrotic placental material [31, 32]. Genetic and functional alterations in ficolins should be investigated as risk factors for lupus flares in pregnant women.

A recent study has identified a new mechanism by which IFN-*α* induces an antiangiogenic milieu, increasing the sensitivity of endothelial cells to soluble vascular endothelial growth factor (VEGF), also known as fms-like tyrosine kinase-1 (sFlt1), and suggesting that elevated IFN-*α* may contribute to the pathogenesis of preeclampsia in some SLE pregnancies [33].

SLE and pregnancy-induced hypertension (PIH) share histological findings in the placenta, complement dysregulation, and fetal outcomes. Placentas of patients with SLE (50%) and PIH (35%) showed a higher *H*-score (range 0–300) for C4d immunoreactivity than control cases with linear staining on the membrane of the syncytiotrophoblast. High C4d groups were significantly associated with low-placental weights and low birth weight in both SLE and PIH. C4d might be utilized as a biomarker for a subsequent risk for IUGR and control during the gestation period in pregnant SLE patients [34].

Pregnancy induces substantial changes in hormone levels [35]. High prolactin (PRL) levels seem to be associated with active SLE during pregnancy. PRL, a cytokine, can influence immune responses. High PRL has been found in 20–30% of SLE patients and seems to be associated with clinical activity during pregnancy [36].

Anti-PRL autoantibodies have been found in 13.1% of pregnant SLE patients. Pregnant SLE women with anti-PRL autoantibodies had fewer adverse outcomes of pregnancy. This suggests PRL may play a role in pregnancy outcomes [37].  Table 1 summarized the main hormonal and immune responses are present in normal pregnancy and lupus pregnancy.

## 3. Lupus Flares during Pregnancy

An important aspect of pregnancy in SLE patients is the risk of disease flares. It is not simple to quantify the incidence of these complications because many clinical studies were performed using individual definitions of flare. Recently, efforts have been made to create a “pregnancy-version” of existing activity indexes, such as the ECLAM, SLEDAI, SLAM, and LAI, aimed at making studies more comparable [38]. The British Isles Lupus Assessment Group (BILAG) 2004-Pregnancy index has been shown to be reliable for the assessment of disease activity in pregnant SLE patients [39].

The results of prospective, controlled observational studies show some discordancy: some studies found that women are at increased risk of lupus flares when pregnant, while other studies found the rate of flares was unchanged as compared to nonpregnant SLE patients [38]. This discrepancy may be explained by disease heterogeneity, the limited number of patients enrolled in SLE-pregnancy studies, the lack of homogeneous criteria for defining lupus flares, and the different SLE treatments used during pregnancy. In addition, several manifestations secondary to pregnancy may be wrongly attributed to lupus flares, including arthralgia, myalgia, facial and palmar rash, hearing loss, and edema in the face, hands, and lower limbs. Likewise, serological abnormalities used to define lupus flares may be physiologically altered during pregnancy, that is, complement and erythrocyte sedimentation rate.

A disease flare may occur at any time, but there may be a trend towards flares in the third trimester. As the timing of flares is unpredictable, regular follow-up is indicated throughout pregnancy and postpartum. There seems to be a consensus that the risk of flares depends on the level of maternal disease activity in the 6–12 months before conception and is higher in women with repeated flares before conception [38], in those who discontinue useful medications (in particular hydroxychloroquine) [40] and, in particular, in women with active glomerulonephritis at conception [41].

Poor control of disease activity before pregnancy may also have detrimental effects on pregnancy outcomes. A study showed that disease activity in the 6 months before conception was associated with an increase in the rate of pregnancy loss [7]. Patients with a combination of high SLE activity and low complement or positive anti-dsDNA had the highest rate of pregnancy loss and preterm birth [42] and hence the importance of a careful evaluation of the maternal condition before and during pregnancy. In fact, patients who started a pregnancy in a stable remission period and continued on medications experienced fewer flares, which were mostly mild and generally well managed with a temporary increase in the prednisone dose [40].

In a recent study, 132 pregnancies in 96 SLE patients were prospectively followed by monthly clinical and laboratory evaluations. Predictors of SLE flares during pregnancy were identified by stepwise logistic regression analysis. Maternal lupus flares occurred in 57% of pregnancies and were best predicted by the number of flares before conception.  Table 2 depicts the types of flares and the respective predictors. The same features of previous manifestations were the best predictors of further manifestations: dermatological flares by previous skin rash, renal flares by previous nephritis, and haematological flares by previous haematological abnormalities [43].

Thrombocytopenia in SLE during pregnancy indicates higher disease activity, severe organ damage, early onset preeclampsia, and higher pregnancy loss. Central nervous system (CNS) lupus in pregnancy represents an especially severe manifestation of SLE and may involve great maternal and fetal risks [44]. Compared with nonpregnant active female SLE patients, active pregnant-related lupus, including new-onset lupus and flare lupus, had a higher incidence of renal and hematological involvement but less mucocutaneous and musculoskeletal involvement [44].

### 3.1. Lupus Nephritis (LN)

The presence of renal disease (of any cause) is a significant risk factor for obstetric complications [41, 45–47], and even small reductions in the glomerular filtration rate (GFR) may increase the chance of pregnant women to develop PE [48]. LN is among the findings that most often induces increased morbidity and mortality during pregnancy. Patients with LN more frequently use high doses of corticosteroids and immunosuppressive agents have a higher frequency of severe infections and hospital admission and also have an increased mortality rate [49]. Active LN shows positive association with premature delivery, increased frequency of hypertension, and of PE [50].

To distinguish clinical indicators of LN activity from pregnancy physiological manifestations and those related to PE can be a challenge. In the first trimester of pregnancy, maternal systemic circulation suffers remarkable physiological vasodilation and relaxin, a hormone produced by the corpus luteum, is a major contributor. One of the results of these changes is a physiological elevation of the GFR and consequent serum creatinine reduction, so values of 0.9 mg/dL may suggest an underlying renal disease requiring further investigation. Protein excretion in the urine is also increased and rates equal to or above 300 mg/24 hours are considered pathological [51, 52]. Therefore, during pregnancy patients with LN may have isolated elevation of proteinuria that is not necessarily indicative of active nephritis.

The patients with LN have 2-3 times higher chance of flare when compared to patients without LN, both systemic and renal disease activity [53]. On the other hand, the use of azathioprine during pregnancy by patients with LN was associated with a lower frequency of flare [54].

Varying results have been reported on pregnancy outcomes in SLE women with preexisting LN. The rate of successful pregnancies ranged between 65% and 92% and the incidence of flares ranged between 8% and 30% [41, 42, 48, 55–57]. A systematic review and meta-analysis of pregnancy outcomes showed a significant association between active LN and both the onset of maternal hypertension during pregnancy and the rate of premature birth [50].

PE, which is more common in women with SLE, detected in up to 20% versus 7.6% in healthy pregnant controls. This obstetric complication have clinical and laboratory manifestations that can be fully superimposable to the active LN, which is in turn a risk factor for the development of PE [50, 51, 58]. Furthermore, patients with LN tend to develop PE earlier compared with women with SLE without nephritis [48].

One of the most complex and challenging aspects during a pregnancy of a woman with lupus is the precise characterization of the LN activity and the differentiation of PE. In both complications hypertension, proteinuria and edema are present. The differential diagnosis is essential, as the treatment varies significantly: in PE, delivery should be considered, while immunosuppressive drugs should be administered to patients with SLE nephritis. LN is likely to be associated with positive anti-dsDNA antibodies (especially in high titers), serum complement consumption, and dysmorphic hematuria and/or red blood cell cylinders. In this scenario, the probable diagnosis is a proliferative kidney disease [59]. During clinical evaluation, the onset of fever, presence of discoid or subacute cutaneous lupus lesions, vasculitis, oral ulcers, polyserositis, lymphadenomegaly, positive direct Coombs, myocarditis, and pneumonitis also indicate lupus flare. In contrast, if the gestational age is greater than 22 weeks, with no signs of SLE activity and hyperuricemia is present, we can state the diagnosis of PE with relative precision. Some features, if present, may help to distinguish between the two conditions (Table 3). However, in many circumstances the patients' manifestations are not complete.

Renal biopsy is a valuable research tool for accurate characterization of LN, but it is usually avoided during pregnancy considering technical difficulties of this procedure in pregnant women. Either way, a recent study found that renal biopsy provides useful information for the management of patients with LN during pregnancy [60].

Complementary elements, such as some angiogenic (VEGF and placental growth factor (PlGF)) and antiangiogenic cytokines (sFlt-1 and soluble endoglin), may help to differentiate between LN and PE [61]. Recent papers have shown that patients with nonpregnant LN, regardless of flares, have increased values of angiogenic cytokines compared to women without SLE, [62] while levels of the antiangiogenic cytokine sFlt-1 is higher in women with active LN compared to patients without flares [63]. Patients with PE have progressive reduction of the amounts of angiogenic cytokines, the opposite expression identified in patients with SLE and LN, and also rising antiangiogenic titers. These results are preliminary and more research is needed before we can employ these citokines to differentiate between these two conditions in routine clinical practice.

## 4. Maternal Outcome

### 4.1. Organ Involvement and Pregnancy

Patients with a high degree of irreversible organ damage are more likely to suffer complications or worsening of previous damage during and after pregnancy [64]. LN is a major manifestation of SLE and, therefore, it is common to have a pregnancy in SLE women with a biopsy-proven diagnosis of renal disease. SLE patients with active LN are at higher risk for pregnancy complications than those without and should be advised against pregnancy until they have renal remission of at least 6 months, and if possible, 12–18 months, according to recommendations [55]. Women with quiescent disease (proteinuria <500 mg/day and inactive urinary sediment) and unaffected renal function are at reasonably low risk during pregnancy but should be closely monitored.

In normal pregnancy, the glomerular filtration rate increases by 30–50% and creatinine clearance rises to <100 mL/min, causing a decrease in serum creatinine. Tubular reabsorption of protein is decreased during pregnancy, and this is why an increase in the normal proteinuria level to 150–180 mg/24 h is possible. However, new-onset proteinuria >300 mg/24 h may be considered pathological in pregnant patients without proteinuria at baseline. Varying results have been reported on pregnancy outcomes in SLE women with preexisting LN. The rate of successful pregnancies ranged between 65% and 92%, and the incidence of flares ranged between 8% and 30% [7, 41, 42, 48, 55–57].

A systematic review and meta-analysis of pregnancy outcomes showed a significant association between active LN and both the onset of maternal hypertension during pregnancy and the rate of premature birth. A history of LN was also associated with preeclampsia. It should be noted that during pregnancy renal flare may determine further loss of kidney function in the short and the long term, with potential accelerated progression to end stage renal disease [50].

Apart from renal involvement, other organs may be negatively influenced by pregnancy. Patients with restrictive pulmonary disease may worsen during pregnancy due to thoracic compression by the growing uterus. Likewise, women with cardiac disease may be at risk of heart failure due to volume overload caused by the normal increase in circulating volume [65, 66]. Contraindications to pregnancy are shown in the following part.

Contraindications to pregnancy in SLE patients are as follows: severe pulmonary hypertension (estimated systolic PSAP > 50 mmHg or symptomatic); advanced heart failure; severe restrictive lung disease; moderate/severe chronic renal failure (creatinine > 2,8 mg/dL); current use of cyclophosphamide, mycophenolate mofetil, methotrexate, leflunomide, statins and angiotensin converting enzyme inhibitor; active renal (24 h urinary protein > 0.5 g) or CNS disease in the past 6 months; recent major thrombosis (<2 years).

Cardiovascular disease (CVD) is a leading cause of morbidity and mortality in SLE. Whether pregnancy acts as a vascular stress test that unmasks endothelial vulnerability to injury that manifests as PE or whether PE itself causes damage that is responsible for future CVD is not clear. A recent preliminary study found that SLE patients with a history of PE had an almost fourfold increase in the rate of subclinical CVD, although there was no association with the presence of plaque [67].

### 4.2. Morbidity and Mortality

A recent US study of pregnancy-related admissions, using information derived from discharge codes, found that women with SLE may be at increased risk of serious medical complications and mortality in comparison with non-SLE pregnant women [58]. At conception, SLE patients had more comorbidities than healthy women. Specifically, SLE patients had more pregestational diabetes, arterial hypertension, pulmonary hypertension, renal failure, and thrombophilia [58]. Moreover, SLE patients tended to become pregnant at an older age compared with the general population. Even after adjustment for increased age, the risk of maternal complications in women with SLE remained higher than in healthy pregnant women. There was an estimated 2–4-fold increase in the rate of caesarean section, PE, and eclampsia, especially in women with preexisting hypertension and/or renal insufficiency taking high-dose prednisone. The risk of sepsis and pneumonia was greatly increased, due to both disease-related immune dysregulation and immunosuppressive therapy. The risk of sepsis and pneumonia was greatly increased, due to both disease-related immune dysregulation and immunosuppressive therapy [58]. Hematological complications requiring transfusion, such as postpartum haemorrhage, antepartum bleeding, anaemia at delivery, and thrombocytopenia were more common in SLE patients [68].

The risk for both venous thromboembolism and stroke was 6.5- fold higher than that of healthy pregnant women, and the excess maternal mortality rate was estimated at 20 times higher than in the general population. These data, collected using the discharge diagnosis, may be not comparable with those derived from tertiary referral centres in which careful multidisciplinary management of pregnant women with SLE allows better maternal and fetal outcomes. However, the data underline the potential risk of increased maternal morbidity and mortality and suggest the need for a high level of vigilance during SLE pregnancies [69]. Several clinical and biochemical factors have been associated with adverse maternal and fetal outcomes in patients with SLE as shown in the following part.

*Clinical, Serological, and Biochemical Factors Associated with Adverse Maternal and Fetal Outcomes in SLE*

Clinical factors are as follows: active disease within 6 months prior to conception and during pregnancy, SLE onset during pregnancy, active lupus nephritis or chronic kidney disease (creatinine > 2.8 mg/dL), maternal hypertension, previous fetal loss.

Serological factors are as follows: antiphospholipid antibodies, anti-double-stranded DNA antibodies.

Biochemical factors are as follows: low complement levels, proteinuria, thrombocytopenia.

Catastrophic antiphospholipid syndrome (CAPS) is a rare complication that occurs in less than 1% of patients with APS but is a life-threatening variant of that thrombophilia. The analysis of 409 catastrophic events included in the CAPS Registry showed that nineteen (4.6%) of were presented during pregnancy or puerperium and nine (47%) of those patients also had SLE [70].

## 5. Pregnancy and Neonatal Outcome

### 5.1. Pregnancy Outcome

Despite major improvement in the last decades, the risk of obstetric and neonatal complications in SLE pregnancy is greater than in general population. It has been estimated that women with SLE have fewer live births compared with the general population, in particular those with high disease activity [17]. Maternal lupus activity and the presence of concomitant antiphospholipid syndrome (APS) were found to be associated with major obstetrical complications [7, 17], being estimated that about 20% of pregnancies in women with SLE end with a fetal wastage [10].

The risk of PE, IUGR, fetal loss, and preterm delivery is greater in patients with SLE compared to general population, especially in those with active nephritis [71]. In a systematic review of 2751 pregnancies in 1842 patients with SLE, reactivation of the disease was identified in 25.6% of cases, hypertension in 16.3%, nephritis in 16.1%, PE in 7.6%, eclampsia in 0.8%, and prematurity in 39.4% of births. There was a positive association between preterm birth and active nephritis and between hypertension and active nephritis or nephritis history [50]. The PROMISSE study, a multicenter study of large-scale, reported that 15% of SLE patients without severe disease developed PE, rising to 22% if there were also positive aPL [68].

The EUROAPS registry reported on the obstetrical results of 247 women recently. Recurrent first trimester miscarriage followed by fetal loss was the most common obstetric morbidities in this mostly Caucasian cohort. Several types of obstetric complications appeared in 52% of the cases; most did not lead to fetal death. Prematurity was the most common finding (47%), followed by stillbirth and fetal loss (22.5%), miscarriage (16%), fetal growth restriction (14%), and early (13%) and late onset PE (12%). All laboratory categories of aPL distributions are represented in the registry. The presence of lupus anticoagulant (LAC), isolated or in combination with anticardiolipin (aCL) and/or anti-beta2-glycoprotein I (anti-*β*2GPI), was the strongest marker related to poor obstetric outcomes. Low complement levels were found in almost 50% of the complicated cases, showing classic complement pathway activation. Maternal and fetal outcomes were good when the currently accepted treatment was given; however, no effort should be spared to improve these recommendations. Thrombosis and progression to SLE in mothers with obstetric APS were uncommon compared with “classical APS.” Showing that differences between classical APS and obstetric APS may exist, the authors conclude that more studies have to be done to understand the laboratory subsets and obstetric outcome [72]. With the use of the Global APS Score (GAPSS) it was demonstrated that, in the SLE population, the presence of LAC is the strongest risk factor for thrombosis. In addition to that, the higher the number of positive tests (single versus double or triple positivity), the stronger the association with thrombosis [73].

The review of 76 pregnancies in 63 lupus patients at the Hospital Universitário Pedro Ernesto-UERJ found similar results. Fifteen percent of patients developed PE, 30% hypertension, and 27% of fetuses had chronic fetal distress and 14 patients needed to increase the dosage of corticosteroids during pregnancy. The birth occurred in a mean gestational age of 35 weeks and fetal death was more frequent in patients with nephritis when compared to patients without nephritis (37% versus 12.2%) [47]. Similarly, Clowse et al. reported that, regardless of clinical activity, both low complement and presence of anti-DNA in the second trimester were associated with higher rate of fetal loss and preterm delivery. When combined with clinical activity, the presence of these parameters was more strongly predictive of fetal loss and prematurity [42].

In a cohort of 96 patients, Borella et al. evaluated prospectively 132 pregnancies and reported 12% of PE, 8% of premature rupture of membranes, and 12% of preterm premature rupture of membranes. Twenty-two percent of live births were preterm, while 17% of the patients suffered pregnancy loss. Fetal loss was best predicted by hypertension at conception, while preterm birth was associated with coexistence of APS and the title of anti-ds DNA before pregnancy. Interestingly, the presence of APS was not associated with pregnancy loss, the fact that could be explained by the use of low dose aspirin and heparin in patients with APS and/or positive aPL [43].

As it can be noted, there is a high incidence of preterm delivery. It can be spontaneous, usually related to premature rupture of membranes, or iatrogenic as an option to protect the health of the mother and/or the fetus, like in cases of PE or fetal distress [74]. Other risk factors for preterm delivery include disease activity prior to and during pregnancy (including serological activity, i.e., high title anti-DNA antibodies, low serum complement levels) [42], higher prednisone dose, and hypertension [7]. Neonatal death, long-term medical complications, and cognitive impairment are more frequent in babies born before 28-week gestation [75], so voluntary interruption of pregnancy before this point should be carefully discussed between physicians and the family. Regarding the birth of babies with low weight (<2500 g) or small for gestational age (birth weight less than the 10th percentile for gestational age), these conditions are more common in SLE pregnancies, ranging from 6 to 35% [76]. This finding is not surprising because placental insufficiency, which is frequent in lupus pregnancies [77], leads to IUGR and to the birth of small for gestational age infants.

### 5.2. Neonatal Lupus Syndromes

Neonatal lupus syndromes refer to a clinical spectrum of cutaneous, cardiac, and systemic abnormalities observed in newborn infants whose mothers have Ro/SSA and La/SSB autoantibodies. The condition is rare, usually benign and self-limited, but may sometimes have serious consequences. It is due to passive transfer of anti-Ro/SSA and/or anti-La/SSB antibodies in some babies of mothers with autoimmune disease [78].

These autoantibodies may damage the developing tissue and increase the risk of bearing infants with neonatal lupus erythematosus (NLE). Approximately 98% of affected infants have maternal transfer of autoantibodies against Ro/SSA, La/SSB, and, less commonly, U1-RNP. However, only 1-2% of mothers with these autoantibodies have neonates with NLE, regardless of whether the mothers are symptomatic or not [79].

The diagnosis is usually made based on the clinical features and the demonstration of neonatal lupus-associated antibodies in the serum of the mother or affected infant. The most common clinical manifestations of NLE are, in decreasing order of frequency, dermatologic, cardiac, and hepatic abnormalities. Some infants may also have hematologic, neurologic, or splenic abnormalities. The most serious complication in the neonate is complete heart block (CHB), which occurs in approximately 2% of such pregnancies. CHB can be devastating for these babies: over 60% require a pacemaker (and recurrent changes as the child grows), 10% will develop cardiomyopathy late after birth despite pacemaker placement, and the 10-year mortality rate is 20–35% [79].

### 5.3. Pregnancy in Mothers with Anti-Ro Antibodies

The high risk of CHB is the most important issue related to the presence of anti-Ro antibodies during pregnancy. CHB normally develops between 18 and 24 weeks of gestation and is usually preceded by lesser degrees of conduction delays, which may be reversed with early treatment [80]. However, conduction abnormalities may progress very rapidly and CHB is often the first rhythm abnormality detected. Various tools have been developed for the early detection of lesser degrees of heart block, including fetal Doppler echocardiography, fetal kinetocardiogram, and transabdominal fetal electrocardiogram [81, 82], although fetal Doppler echocardiography remains the most commonly used modality. All fetuses exposed should be monitored weekly between 16 and 26 weeks of gestation and biweekly thereafter [83]. Detection of an early conduction defect such as a prolonged PR interval should be considered a danger signal.

Although some early blocks are transient, progression to CHB remains unpredictable. Prophylactic treatment should be discussed if the PR interval remains persistently prolonged. Maternal administration of fluorinated corticosteroids has shown benefits on fetal survival in some studies. However, the results are not consistent and the benefits must be weighed against the higher risk of IUGR and preterm birth [81]. Treatment of established CHB remains even more unsatisfactory. Improved fetal outcomes were reported after transplacental treatment with dexamethasone and beta-adrenergic stimulants in one study, but these findings were not replicated [84]. Hydroxychloroquine during pregnancy, on the other hand, reduces the risk of cardiac NLS in at-risk fetuses [85, 86].

The risk of recurrence of CHB in subsequent pregnancies after an affected pregnancy is much greater, but hydroxychloroquine reduced this risk by 65% in one study [85]. Open label data also showed beneficial effects for intravenous immunoglobulin (IVIG), but two large randomized controlled trials showed negative results [87, 88]. The study design, the dose of IVIG used, and the differing composition of IVIG may have contributed to the negative outcomes [89]. In summary, there is no satisfactory current treatment for established CHB [90].

There is consensus on the prognostic factors of poor outcomes associated with neonatal CHB: detection of CHB at gestational age < 20 weeks, ventricular rate < 50 bpm, fetal hydrops, changes in the fetal echocardiogram, carditis, impaired left ventricular function, endocardial fibroelastosis, and a maternal diagnosis of SLE or Sjögren's syndrome [91, 92].

## 6. Prenatal Care

**Figure 2:**
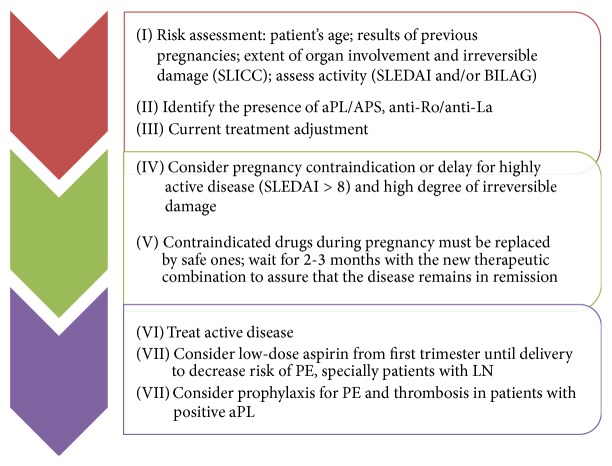
Counselling and pregnancy planning for patients with lupus.

Ideally, all patients with SLE that desire to get pregnant should have a preconception visit, when the physician evaluates the risks associated to the pregnancy, medications that are contraindicated during pregnancy and if the patient is in the best moment to get pregnant according to underlying disease activity and complications (Figure 2). Planned pregnancies have demonstrated reduced flare rates and better obstetric outcomes in women with SLE [1].

After conception, antenatal management of pregnant patients with SLE requires close collaboration between rheumatologist and obstetrician [1]. The woman with SLE who gets pregnant should be submitted to clinical and laboratory evaluations during the first visit, in order to identify SLE disease activity and situations that increase risk of fetal complications. Recommended visits, laboratory tests, and ultrasound evaluations are listed on the following part.

*Visits, Laboratory Tests, and Ultrasound Evaluation Recommended during Prenatal Care of Patients with Lupus*

*Visits*

- Obstetrician visits are as follows:
  - monthly until 20 weeks,
  - every two weeks until 28 weeks,
  - weekly after 28 weeks until delivery.
- Rheumatologist visits are as follows:
  - ideally, a rheumatologist should support the obstetrician during prenatal care;
  - if not possible, the rheumatologist should see the patient every 4–6 weeks.

*Laboratory Tests*

- First visit tests are as follows:
  - complete blood count, platelet count, prothrombin activation time, and partial thromboplastin time;
  - lupus anticoagulant; anticardiolipin antibody IgG and IgM; and anti-*β*2 glycoprotein I IgG and IgM (which must be repeated in 12 weeks if positive);
  - anti-Ro/SS-A, anti-La/SS- B, anti-Sm, and anti-RNP;
  - blood glucose, BUN, creatinine, uric acid, AST, and ALT;
  - anti-DNA, C3, C4, and CH50;
  - urinary sediment; 24-hour proteinuria or protein/creatinine ratio in a single urine sample; dysmorphic research erythrocyte in urine, creatinine clearance and urine culture.
- Quarterly visit tests are as follows:
  - complete blood count, platelet count;
  - anti-DNA, C3, C4, and CH50;
  - blood glucose, BUN, creatinine, uric acid, AST, and ALT;
  - 24-hour proteinuria or protein/creatinine ratio in a single urine sample if preeclampsia or lupus nephritis is suspected, as well as research erythrocyte dysmorphism.

*Ultrasound and Doppler Velocimetry Studies. *They include the following:

- monthly after 24 weeks: evaluation of fetal growth, amniotic fluid, and umbilical artery (fetal-placental flow),
- uterine artery evaluation at 24 weeks: screening tests for preeclampsia and intrauterine growth restriction.

Intervals of the visits and frequency of laboratory tests may be smaller in case of disease activity or suspected PE.

Laboratory tests should be interpreted in the light of the knowledge of the changes imposed by pregnancy itself. Despite being used as a marker of inflammatory disease activity, erythrocyte sedimentation rate (ESR) rises by pregnancy itself, so it should not be used in pregnant women with SLE. Serum complement levels tend to increase during pregnancy and its fall should be assessed relative to a baseline test. Incidentally thrombocytopenia may occur in about 10% of pregnant women and it becomes difficult to distinguish from lupus activity. The urinary excretion of proteins, which normally rises during pregnancy, can reach about 300 mg/24 hours without having clinical significance [1]. Also, pregnant women generally tend to have urinary tract infections, and the use of immunosuppressants agents may inhibit cellular deviation and leukocytosis.

Renal function should be assessed even in patients without nephritis history as it can be asymptomatic or start during pregnancy, as well as the need for a baseline for comparison in the case of kidney injury during the course of pregnancy. Hepatic involvement is uncommon in patients with SLE, but the evaluation of their role is required especially in patients taking azathioprine because of its hepatotoxicity, with repeat tests at least every 3 months.

Regarding the mentioned antibodies, aPL (LAC, aCL, and anti-*β*2GPI) is markers of adverse pregnancy outcomes and may be helpful in the case of a thrombotic event during pregnancy. It should be remembered that the presence of aPL in lupus patients without obstetric (recurrent spontaneous abortion, fetal loss) or thrombotic events (deep vein thrombosis, arterial thrombosis) does not provide the diagnosis of APS or even justifies the prescription of heparin to these patients. Most of authors recommend the use of low dose aspirin during pregnancy for patients with SLE and positive aPL [93].

Lupus flares are likely to be associated with hypocomplementemia and increased titers of anti-DNA antibodies; in comparison, complement levels are usually (but not always) increased in patients with preeclampsia. The anti-Ro/SSA and anti-La/SSB are responsible for neonatal lupus and anti-Sm is the antibody specific for SLE. Patients with positive anti-Ro/SSA or anti-La/SSB should perform fetal echocardiography weekly from 16 to 26 weeks and biweekly thereafter [1]. ANA, anti-Ro/SS-A, anti-La/SS-B, anti-Sm, and anti-RNP do not change with disease activity and therefore do not need to be repeated later.

Current standard of care in SLE pregnancy includes Doppler studies of uterine arteries and umbilical artery, which are helpful to assess placental function and to exclude the occurrence of complications such as PE and fetal distress [40]. Uterine Doppler studies are useful as screening test for PE and the 24th week is the best moment for the evaluation. Abnormal uterine artery Doppler studies have been identified in patients with SLE with posterior fetal loss, PE, IUGR, and preterm labor [94, 95].

Umbilical Doppler ultrasound gives a more accurate definition of the placental function, showing various degrees of impairment such as increased resistance, absent or even reverse diastolic flow, which is a clear sign of placental insufficiency and fetal distress [41, 96]. Follow-up with monthly ultrasound and Doppler velocimetry studies starting at 24 weeks to assess fetal growth, amniotic fluid, and fetal-placental flow is recommended considering the high incidence of fetal growth restriction and chronic distress [96]. Alterations of umbilical artery Doppler velocimetry should be managed similarly to those patients without SLE. Normal evaluation of these tests has a high negative predictive value for fetal death. It is important to notice that these previously cited fetal complications may be related to the presence of aPL.

## 7. Treatment

As already discussed, SLE can reactivate during pregnancy, situation that requires proper handling of antirheumatic drugs. The use of hydroxychloroquine during pregnancy is accepted and recommended by members of the American College of Rheumatology and the European League against Rheumatism (EULAR) [97, 98]. Hydroxychloroquine use during pregnancy in patients with SLE reduces the number of flares and hypertensive disorders [99], so its use should be continued during pregnancy or prescribed for those that are not using. Its use during pregnancy is safe, without reported malformations, growth restriction and ocular, auditory, or neurological toxicity in exposed fetus [2, 40]. Chloroquine has smaller data when compared to hydroxychloroquine, but no long-term sequel was also demonstrated [100]. In addition, a systematic review did not report any ocular abnormality in children born from mothers treated with chloroquine or hydroxychloroquine during gestation [101]. Both are secreted in breast-milk, but there was no report of adverse effects in breastfed children whose mothers used hydroxychloroquine [100].

Corticosteroids represent the treatment of choice in cases of reactivation of SLE during pregnancy and can be used as prednisone or prednisolone. These compounds are inactivated by the enzyme 11-*β*-hydroxysteroid dehydrogenase 2 and reduce fetal exposure to approximately 10% of maternal dosage [102]. Therefore, the use of these drugs does not replace the use of betamethasone or dexamethasone when they are indicated for fetal lung maturation. The dose should be the minimum necessary and depends on the impaired organ, using the same criteria recommended for nonpregnant women, with great parsimony as possible in order to minimize adverse events. The use of doses above 10 mg/day of prednisone is associated with increased risk of developing arterial hypertension, dyslipidemia, fluid retention, and maternal hyperglycemia. Intravenous pulses of methylprednisolone can be used safely if indicated for severe activity [40]. Corticosteroids do not enter breast milk in large quantities and there is no contraindication to breastfeeding in women who are on corticosteroid therapy [103]. Women that are breastfeeding and using higher doses of corticosteroids should wait 4 hours after taking the pill to breast-feed, reducing the drug concentration in the breast-milk [100].

There is no evidence that the prophylactic use of corticosteroids in SLE pregnant women without active disease prevents exacerbations during pregnancy, so this conduct is considered inappropriate.

The nonsteroidal anti-inflammatory drugs can be administered to management arthralgia or serositis in the lowest dose possible for a short time and it is recommended to be completely discontinued after the 32th week. After this time, there is high risk of fetal and maternal hemorrhage in addition to fetal renal dysfunction, oligodramnia, and premature closure of arterial duct [100].

Azathioprine in doses up to 2.5 mg/kg/day remains one of the therapeutic resources available in cases of SLE activation during pregnancy, being the immunosuppressant of choice for the treatment of severe maternal disease or refractory to isolated use of corticosteroids. This immunosuppressant and steroid sparing agent is not associated with teratogenicity in humans, as the fetal liver is not capable of metabolizing azathioprine into its active form [100], although teratogenicity has been reported in animal studies [104]. The treatment with azathioprine is compatible with breastfeeding, with no risks for the child [100].

Cyclophosphamide, mycophenolate mofetil, leflunomide, and methotrexate have teratogenic effects and should not be used during pregnancy [104]. Mycophenolate mofetil should be stopped at least 6 months prior to conception and changed to azathioprine, with a small risk of flare during this period [54].  Table 4 summarizes recommendations for the use of medications during pregnancy and lactation of patients with SLE.

## 8. Contraception and Assisted Reproduction

Pregnancy outcomes are optimized when pregnancy is planned in patients with SLE and APS. Contraception in SLE/APS patients goes beyond the simple need to avoid unwanted pregnancies. Several conditions may require effective contraception: early stage of the disease, very active disease, severe organ involvement or damage, and the use of embryotoxic/foetotoxic drugs. Therefore, contraceptive counselling is essential in clinical rheumatology, although many women still do not receive adequate information in clinical practice [105].

Effective contraception is underused in patients with rheumatic diseases. In a study of 97 SLE patients at risk of pregnancy, 55% had unprotected sex occasionally and 23% ‘‘most of the time” [106]. In another study of 86 patients, 55% of those using contraceptives regularly were using less-effective barrier methods only, even when being on teratogenic medications [105].

A common misconception among women with SLE is that they “cannot use birth control,” since the “classical” estrogen-containing pill is generally contraindicated. The message should be that women with SLE should be considered good candidates for many contraceptive methods, including hormonal contraceptives, and the most suitable choice should be made individually [107].

The three main types of contraceptives are barrier methods, intrauterine devices (IUD), and hormonal methods. Barrier methods are an effective, cheap method of preventing pregnancy, and sexually transmitted disease. However the unintended pregnancy rate remains at around 17% for condoms and diaphragms. IUD are available in nonmedicated or medicated (progesterone) forms. With typical use, the rate of unplanned pregnancy is around 2%. Complications include irregular bleeding after placement, the risk of expulsion (5% falling out over the 5-year life of the device), and the risk of infection after insertion that may lead to pelvic inflammatory disease (PID). Current IUD devices are actually safer in high-risk groups [108], but the risk of infections should be continuously monitored. It may be preferable to use IUD in patients with a single sexual partner and mild treatment (no immunosuppressive drugs, prednisone < 10 mg per day). The use of estrogen-containing oral contraceptives (OC) has been discouraged due to initial reports of lupus flares due to hormonal treatment and subsequently supported by growing experimental evidence of the role of estrogens in the pathogenesis of SLE [109]. However, two recent randomized clinical trials [110, 111] supported the safety of low dose combined OC in a well-defined population of stable SLE patients with inactive or stable active disease with respect to the risk of lupus flares. However, the presence of aPL remains a major contraindication to combined OC due to the increased risk of thrombosis. Progestin-only preparations (daily oral pill, depot medroxyprogesterone, and subcutaneous implants) do not appear to increase immune activity and are not associated with increased rates of flares, and the dose of progestin does not increase the risk of thrombosis; a recent large study in SLE patients showed good gynaecological tolerability (low rate of discontinuation due to breakthrough bleeding or hypoestrogenia) [112]. A major concern about the use of progesterone is its effects on bone health; however, the reduction in bone mineral density has been shown to be reversible after discontinuation of treatment [113].

Women affected by SLE have an overall fertility rate similar to that of the normal obstetric population, with a mean of two live births [114]. However, there are some conditions in which fertility may be impaired [115], like presence of chronic renal failure [116] and use of cyclophosphamide. The risk of infertility is related to the cumulative dose of the drug and the patient's age, with older women having a lower ovarian reserve at higher risk for premature ovarian failure. Protection of ovarian function can be provided by treatment with gonadotropin-releasing hormone analogues [117]. Women should be informed that NSAIDs may inhibit ovulation and that these should be stopped at day 8 of the menstrual cycle when they want to conceive [118].

Whatever the cause of infertility, whether being disease-related or not, patients with SLE may need medically assisted reproductive techniques (ARTs). The technique most-frequently used is in vitro fertilization and embryo transfer (IVF-ET), which requires ovarian stimulation for oocyte pick-up. Ovarian stimulation may create concern in SLE/APS women for several reasons, both theoretical and due to results from small studies [119]: (1) high dose estrogens may induce a disease flare; (2) the enhanced hormonal milieu may increase the risk of thrombosis, especially in women with aPL; (3) these complications may become life threatening in the case of ovarian hyperstimulation syndrome; (4) there may be a trend towards a worse prognosis for both pregnancy rates and live-birth rates after ARTs. Antithrombotic prophylaxis should be carried out in women with aPL, with particular attention paid to women with a prior thrombosis [120].

ARTs may be considered for patients with SLE and APS as these procedures do not appear to increase the risk of disease flares or thrombosis. In addition, the presence of aPL does not independently predict the outcome of IVF pregnancies. As with natural pregnancies, it is recommended that candidates for ART should have quiescent disease for at least six months prior to attempting to become pregnant, to ensure the best possible outcome for mother and child [121].

Recent studies consistently state that screening ART candidates for autoantibodies and treating for positive findings is not justified [122–125]. The US Practice Committee of the American Society for Reproductive Medicine has published guidelines to this effect [126]. Blindly testing for and identifying these autoantibodies in otherwise asymptomatic women may cause undue anxiety given the prognostic uncertainty of the findings.

## 9. Conclusions

SLE pregnancies are considered high risk condition and recent studies reinforce the importance of planning the pregnancy. As suggested by several authors, the association of SLE and pregnancy, mainly with active disease, especially nephritis, has poorer pregnancy outcomes, with increased frequency of PE, fetal loss, prematurity, growth restriction, and newborns small for gestational age. The differential diagnosis from PE and LN remains a challenge. Antenatal management of pregnant patients with SLE requires close monitoring and collaboration between rheumatologist and obstetrician. With better treatments and preservation of renal function, combined with current recommendations for planning pregnancy during the quiescent period, a better maternal and fetal prognosis can be expected in pregnancies of patients with SLE.

## Figures and Tables

**Table 1:** Hormonal and immune response differences between normal pregnancies.

Immune and hormonal response	Normal pregnancy	Lupus and pregnancy	Clinical manifestation associated
Th17: IL-17	High	Higher increase	Preeclampsia and pregnancy loss

Estradiol and progesterone	Higher in second and third trimester	Lower in second and third trimester	Impaired placental function and fetal loss

IL-6	Low at first trimester but high in the third trimester	Low in the three trimesters	Altered immune regulation from T cell to B cell

IL-10	Low in the trimester but high in the last trimester	High since preconception throughout pregnancy and still postpartum	Continuous B cell stimulation

Treg cells	High	Low number and impaired function	Lupus activity

Chemokines CXCL8/IL-8 CXCL9/MIG CXCL10/IP-10	Low	Higher serum concentrations	Increase pregnancy complications and lupus flares

Ficolin-3	Low	Increase	Haemolysis

IFN-*α*	Low	Higher concentration	Contribution to preeclampsia

C4d	Low	Higher concentration	Low placenta weight and low birth weight

Prolactin	Low	Higher concentration	Lupus activity

IL: interleukin; Treg cells: T regulatory cells; CXCL: chemokine ligand; MIG: monokine induced by gamma interferon; IP-10: interferon gamma-induced protein 10; INF-*α*: interferon alpha; C4d: complement component.

**Table 2:** Type of SLE flare during pregnancy, manifestations, risk factor, and conduction.

Type of SLE flare during pregnancy	Manifestations	Risk factor	Management
Mucocutaneous	High inflammatory rash often sparing the nasolabial folds	Anti-Ro/SSA, previous involvement	Topical corticosteroids or oral prednisone, HCQ

Articular	Arthralgia, arthritis, carpal tunnel syndrome (related to pregnancy edema)	Anti-dsDNA positivity	HCQ, NSAID until 28th week, prednisone throughout

Hematological	Cytopenias	aPL, Coombs, previous involvement	Leukopenia: discard drug-related; hemolytic anemia: high dose prednisone and azathioprine; thrombocytopenia: high dose prednisone and azathioprine may use IVIG and rituximab

Renal	Hypertension, edema, proteinuria	aDNA, low C3 and C4 differentiate between APS microangiopathy and preeclampsia, previous involvement	Immunosuppressive treatment with corticosteroids; pulse-methyl prednisolone

CNS	Wide range including depression and psychosis	CNS manifestation provided pregnancy	Antidepressants; pulse-methyl prednisolone

Vascular	Cutaneous vasculitis	Previous involvement	Prednisone and azathioprine

aPL: antiphospholipid antibodies; APS: antiphospholipid syndrome; CNS: central nervous system; HCQ: hydroxychloroquine; IVIG: intravenous immunoglobulin.

**Table 3:** Features differentiating preeclampsia and lupus nephritis.

Clinical and laboratory features	Preeclampsia	Lupus nephritis
Hypertension	After 20 weeks of gestation	Any time during pregnancy
Platelets	Low-normal	Low-normal
Complements	Normal-low	Rising titres
Anti-dsDNA	Absent or unchanged	Normal to raised
Creatinine	Normal to raised	Normal to raised
Serum uric acid	Elevated	Normal
24-hour urine calcium	<195 mg/dL	>195 mg/dL
Urinary sediment	Inactive	Active
Other organs involved	Occasionally CNS or HELLP	Evidence of active nonrenal SLE
Response to steroids	No	Yes

**Table 4:** Recommendations for the use of medications for pregnancy planning.

Medication	Recommendation for pregnancy planning	Indication during pregnancy	FDA 1979 class	Comment for using during pregnancy	Maternal adverse reactions	Fetal/neonatal adverse reactions	Lactation
Aspirin (low dose)	Start before conception	Obstetric APS	B/C	80–100 mg/d used isolated or in combination with LMWH	Bleeding	No	Allowed

Azathioprine	Without stopping	Used in cases requiring immunosuppression and to save corticoid	D^*∗*^	1–2,5 mg/Kg/d	Liver toxicity and bone marrow toxicity	No	Allowed

Belimumab	Not start during pregnancy Not stop during pregnancy	Arthritis, other inflammatory	C	10 mg/kg IV q2 weeks ×3 doses, Maintenance: 10 mg/kg IV q4 weeks	Reactivation of tuberculosis	No	Not allowed

Cyclosporine A			C	Blood pressure monitor	Increase of blood pressure	Low birth weight	Not allowed

Cyclophosphamide	Stop > 6 months before conception	Not allowed	X				Not allowed

Fluorinated corticosteroids	It will be used in more advanced pregnancy	NLE and maturity of fetal lungs	B	Use betamethasone 12 mg/d for two days	Uncontrolled glycemic	No	Allowed

Hydroxychloroquine	Keep the drug to prevent reactivation of lupus	Lupus, arthritis	N	200–400 mg/d	Maternal skin hyperpigmentation	No	Allowed

Leflunomide	Stop 3–6 months before conception	Not allowed	X				Not allowed

Low-molecular weight heparin	Generally used	Obstetric APS	C	Used in prophylactic dose of 0.5 mg/Kg QID for OBAPS and in full dose 1 mg/Kg BID for thrombotic APS	Osteoporosis, thrombocytopenia	No	Allowed

NSAID	Avoid since it may decrease	Arthritis, other inflammatory	C/D	Avoid in the 3rd trimester	Gastric bleeding	Premature closure of the ductus arteriosus, oligohydramnios	Allowed

Methotrexate	Stop 3–6 months before conception	Not allowed	X				Not allowed

Mycophenolate mofetil	Stop before conception	Not allowed	X				Not allowed

Prednisone/prednisolone	Try to minimize dose due to maternal AEs	Lupus, arthritis, other inflammatory diseases	C	Inactivated in the placenta, minimize dose due to maternal AEs	Increase of blood pressure, osteopenia, gestational diabetes, immunosuppression, grooves.	Cleft lip sporadically Low birth weight Premature birth	Allowed (if more than 40 mg, wait for 3 hours)

Rituximab	Not start during pregnancy Not stop during pregnancy	Arthritis, other inflammatory	C	375 mg/m²/IV q4 weeks	Reactivation of tuberculosis	No	Not allowed

Statins	Stop before conception	Not allowed	X				Not Allowed

ACEi	Stop before conception	Not allowed	D			Fetal death, renal dysfunction and oligohydramnios	Allowed

Tacrolimus		Used in cases requiring immunosuppression	C	0,1–0,2 mg/kg/d	Diabetes, increase of blood pressure, reactivation of tuberculosis	Increase of blood pressure, diarrhea, headache, renal dysfunction	Not allowed

Warfarin	Stop at conception	Not allowed	X				Allowed

AEs: adverse events; APS: antiphospholipid syndrome; OBAPS: obstetric APS; ACEi: angiotensin converting enzyme inhibitor.
